# Endogenous retroviruses Suppressyn and Syncytin-2 as innovative prognostic biomarkers in Acute Myeloid Leukemia

**DOI:** 10.3389/fcimb.2023.1339673

**Published:** 2024-01-11

**Authors:** Jiaxin Shen, Xiaofen Wen, Xueyang Xing, Claudio Fozza, Leonardo Antonio Sechi

**Affiliations:** ^1^ Department of Biomedical Sciences, University of Sassari, Sassari, Italy; ^2^ Department of Hematology, The First Affiliated Hospital of Shantou University Medical College, Shantou, China; ^3^ Department of Medical Oncology, Cancer Hospital of Shantou University Medical College, Shantou, China; ^4^ Department of Medicine and Pharmacy, University of Sassari, Sassari, Italy; ^5^ SC of Microbiology and Virology, Azienda Ospedaliera Universitaria (AOU) of Sassari, Sassari, Italy

**Keywords:** human endogenous retrovirus, acute myeloid leukemia, prognostic model, immune infiltration, bioinformatics

## Abstract

**Introduction:**

Emerging evidence has proven that human endogenous retroviruses (HERVs) play a critical role in the pathogenesis of Acute Myeloid Leukemia (AML), whereas the specific HERVs influencing the prognosis of AML patients have yet to be fully understood.

**Methods:**

In this study, a systematic exploration was achieved to identify potential prognostic HERVs for AML, sourced from TCGA and GTEx database. Differential analysis and functional enrichment studies were conducted using GO, KEGG, GSEA, and GSVA. The ESTIMATE algorithm was applied to explore the immune infiltration of HERVs in AML. A prognostic risk-score model was evaluated with predicted yearly accuracy using ROC analysis.

**Results:**

Two HERVs Suppressyn and Syncytin-2, were identified as promising prognostic biomarkers, with high discrimination ability based on ROC analysis between AML and healthy cohorts from TCGA. Their expression was notably higher in AML patients compared to those in healthy individuals but correlates with favorable clinical outcomes in sub-groups such as white race, lower WBC counts, favorable and intermediate risks, and NPM1 or IDH1 mutation. Suppressyn and Syncytin-2 participated in immune-related pathways and exhibited correlations with multiple immune infiltration cells, such as T cells, mast cells, and tumor-associated macrophages. Finally, we developed a prognostic risk-scoring model combining Suppressyn and Syncytin-2, where a high risk-score is associated with better prognosis.

**Discussion:**

Collectively, our findings revealed that Suppressyn and Syncytin-2 may act as valuable diagnostic and prognostic biomarkers for individuals with AML, while highlighting links between HERV activation, immunogenicity, and future therapeutic targets.

## 1 Introduction

Acute myeloid leukemia (AML) is one of the most common types of hematologic malignancy where myeloid blasts fail to undergo normal differentiation due to clonal expansion(Pollyea et al., 2023). Despite recent therapeutic advances indicated by increasing cure rates, the heterogeneity of AML patients often results in poor outcomes in adults (Short et al., 2020). Advanced knowledge in measurement techniques and pathophysiology, as well as the approval of at least 10 recent therapies have collectively contributed to an updated diagnostic, therapeutic, and prognostic framework of AML(Shimony et al., 2023). Nevertheless, AML remains a disease with highly variable prognosis, underscoring the imperative for novel genetic and molecular predictors.

In recent times, a panel of leukemia cell lines with RNA sequencing-based gene expression data revealed differential expression of human endogenous retroviruses (HERVs) (Engel et al., 2021). Known for million years of assimilating into the human genome, these prehistoric retroviral sequences have now become stable, constituting approximately 8% of our DNA, as compared to the 1-2% attributed to protein-encoding genes(Lander et al., 2001). Structurally, HERVs typically carry three primary coding elements: group-specific antigen (gag), polymerase (pol), and envelope (env), bordered by long terminal repeat sequence (LTRs), all essential for survival and preservation of HERVs(Lindemann et al., 2013). The env, though containing high levels of defects and alternative splicing variants, is being examined for its potential pathogenic properties that could contribute to the intricate etiology of cancer development(Grandi and Tramontano, 2018). The biological gradient of certain HERV products should be crucially evaluated because they might have negligible impact on the host until they are upregulated within a diseased context. One typical example is ERV3-1, the extensively researched HERV-env gene that exhibits upregulation in both blood and bone marrow cells, leading to maintenance of AML phenotype(Nakagawa et al., 2021).

A few recent publications have discovered significant findings that provide convincing evidence of the potential protective roles of HERV-env products. For example, in TRACERx, a large prospective observational cohort study on non-small cell lung cancer, the env-glycoproteins of HERV-K(HML-2) (HGNC: 13915), were demonstrated a dominant anti-tumor antibody target, significantly contributing to amplified B cell responses by immune checkpoint inhibitor, thus enhancing anti-tumor immunity(Ng et al., 2023). Suppressyn, derived from env sequences of HERVH48, could function as a protector from infection by competitively binding to receptors of exogenous viruses in human preimplantation embryos and developing placenta(Frank et al., 2022). More recently, CancerHERVdb provides a consolidated resource for HERV activation, facilitating the identification of cancer drivers, prognostic and risk markers, signals that span across multiple cancer types, and immune therpateutic targets(Stricker et al., 2023a). While these compelling findings imply the potential benefits of a more extensive analysis of HERV signatures in both laboratory and clinical settings, it should be acknowledged that no definitive link has been established between any specific HERV sequence or its expressed products, and the development of cancers. This is due to several confounding factors: inadequate description of individual HERV loci, limited functional knowledge of HERV in healthy and cancerous contexts, and lacking of accurate molecular mechanisms of pathogenesis(Voisset et al., 2008; Liu et al., 2020).

By means of mining public online database, our study focused on investigating specific HERVs that correlated with the progression and immune activity of AML. Besides, we integrate our findings with clinical data and emphasize their potentials for innovative therapeutic targets, aiming to enhance clinical decision-making accuracy and facilitate the assessment of risks and prognosis related to AML. We remain optimistic that our findings on HERVs will contribute to the development of promising strategies that navigate the challenges posed by AML and ultimately enhance patient care and survival rates.

## 2 Materials and methods

### 2.1 Acquisition of AML data

We retrieved mRNA expression profiles and clinical data of AML from TCGA. An overall count of 170 AML patient were included, out of which 139 patients had both clinical data and sequencing data available. We also collected normal bone marrow samples (n=70) from the Genotype-Tissue Expression databases. For analysis, mRNA expression was in HTSeq-FPKM format (level 3) and normalized to TPM reads.

### 2.2 Identification of Differentially Expressed HERVs (DE-HERVs)

The online NetworkAnalyst (https://www.networkanalyst.ca/) (Zhou et al., 2019) was employed to assess various mRNA levels of HERVs in AML, comparing data from the TCGA database with normal samples sourced from the GTEx database. The HERVs were identified following the criteria of P value< 0.05 and absolute log2-fold change > 1.

### 2.3 Prognostic values of Suppressyn and Syncytin-2

Survival analyses were performed by Kaplan-Meier estimates and followed by Cox regression model. Patients were divided into groups using the median expression level of Suppressyn and Syncytin-2 as the threshold.

### 2.4 Functional enrichment analyses of Suppressyn and Syncytin-2

Gene ontology (GO) analysis and Kyoto Encyclopedia of Genes and Genomes (KEGG) pathway analysis were carried out to perform functional enrichment analyses by utilizing R clusterProfiler (version 4.4.4). The significance criteria were set at P value< 0.05 and absolute log2-fold change > 1. The AML cohort obtained from TCGA was divided into two groups based on the median expression scores of Suppressyn and Syncytin-2. The data was visualized using R ggplot2 (version 3.3.6). Moreover, gene set enrichment analysis (GSEA) was conducted using R gplot2 (version 3.3.6) and clusterProfiler (version 4.2.1). Function or pathway terms were considered significantly enriched in a statistical context, if they had an adjusted p-value< 0.05 and a false discovery rate (FDR)< 0.25.

### 2.5 Protein-protein interaction (PPI) network analysis of Suppressyn and Syncytin-2

The Spearman’s correlation analysis was employed to find out whether the diverse expression of Syncytin-2 and Suppressyn correlates with the ten most significant differentially expressed genes (DEGs). The DEGs were used to construct a PPI network using the Search Tool for the Retrieval of Interacting Genes (STRING) online database. The confidence score was considered high when greater than 0.7. Default values were used for all other parameters. Then, the resulting was edited via Cytoscape (version 3.9.1), a software for visualizing networks(Lotia et al., 2013). Ten most significant hub genes were identified using Cytoscape plugin CytHubba(Chin et al., 2014).

### 2.6 Estimation of immune infiltration

Immune Infiltration in AML was computed and analyzed through the ESTIMATE package in R, using ImmuneScore, StromalScore, and ESTIMATEScore(Yoshihara et al., 2013). We assessed the level of immune infiltration by examining 24 immune cells. Single-sample Gene Set Enrichment Analysis (ssGSEA) was able to compute the proportional enrichment of these cells in AML, implemented under the R package GSVA(Bindea et al., 2013). A Spearman’s correlation analysis was used to determine the relationship between Suppressyn, Syncytin-2, and these immune cells. The disparity in immune infiltrates between diverse expression patterns of Suppressyn and Syncytin-2 was evaluated using Wilcoxon rank-sum tests. The connection between immune checkpoints and expression of Suppressyn and Syncytin-2 were further examined by R package ggplot2 (version 3.3.6), aiming to explore their correlation with tumor immunity.

### 2.7 Construction and validation of a prognostic risk-scoring model

A multivariable Cox regression was used to determine the coefficients for DE-HERVs that showed statistical significance in univariable Cox regression. A risk-score formula was constructed as follows:


$$riskscore=\sum_{i=1}^{N}\left(Exp_{i}\times Coe_{i}\right)$$

where N = 5, the Expi denotes the expression value of every five HERV-related genes, and the Coei represents the corresponding coefficient obtained from the multivariable Cox regression. The R package ggplot2 was employed by visualization. Based on the risk scores obtained, AML patients were divided into high- and low-risk groups using the median risk score as a threshold. The overall survival (OS) analysis was conducted between these two groups. We evaluated performance of the model in terms of accuracy of prediction by the receiver operating characteristic (ROC) curves.

### 2.8 Statistical analyses

The significance of diverse expression of Suppressyn and Syncytin-2 was assessed by the Wilcoxon rank-sum test. The association of clinical parameters and Syncytin-2 expression was explored by the Wilcoxon rank-sum test and logistic regression. These analyses were two-sided, conducted by R software version 3.6.3., and statistical significance was defined as P values< 0.05.

## 3 Results

### 3.1 Identification of differentially expressed HERVs (DE-HERVs) in AML cohorts

**Figure 1:**
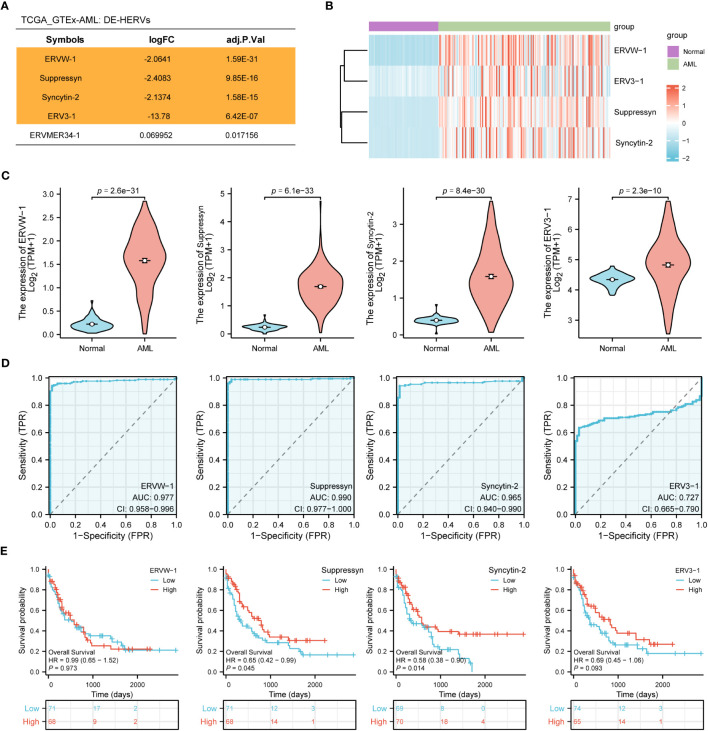
Identification of DE-HERVs **(A)** Five DE-HERVs between AML vs normal from TCGA and GTEx. Orange ones meet the criteria of absolute log2-fold change > 1 and adjusted p-value< 0.05. **(B)** Heatmap of ERVW-1, ERV3-1, Suppressyn and Syncytin-2, with red and blue indicating high and low expression, respectively. **(C)** Expression of ERVW-1, Suppressyn, Syncytin-2 and ERV3-1 in AML vs normal from TCGA and GTEx. **(D)** ROC curves were generated based on the expression of DE-HERVs to distinguish between AML vs normal. **(E)** The OS Kaplan-Meier curves for TCGA patients with diverse expression of four DE-HERVs. DE-HERVs, differentially expressed HERVs; TCGA, The Cancer Genome Atlas; GTEx, Genotype Tissue Expression Project; ROC, receiver operating characteristic; OS, overall survival; HR, hazard ratio; CI, confidence interval.

In the TCGA dataset we collected 173 AML patients, while in the GTEx dataset, we obtained 70 normal samples. Using web-based tool NetworkAnalyst, we identified a total of four DE-HERVs (ERVW-1, Syncytin-2, ERV3-1, ERVMER34-1) based on the cut-off criteria of absolute log2-fold change > 1 and an adjusted p-value< 0.05 (**Figure 1A**). In AML patients, heatmaps and violin plots demonstrated that 4 HERV-related genes were significantly upregulated compared to normal tissue (**Figures 1B, C**). Among them, ERVW-1, Suppressyn and Syncytin-2 displayed excellent predictive performance in distinguishing AML from normal samples, with AUC values of 0.977 (95% confidence interval [CI]=0.958-0.996), 0.990 (95% CI =0.977-1.000) and 0.965 (95% CI =0.940-0.990), respectively, by receiver operating characteristic (ROC) curve analysis (**Figure 1D**).

Next, we applied the Kaplan-Meier approach to observe whether diverse expression of DE-HERVs could affect patient survival in the progression of AML. Patients were classified into high and low expression clusters, stratified according to median values. Our results displayed that increased Suppressyn indicated a significantly improved prognosis for OS (HR = 0.65, 95% CI = 0.42-0.99, P = 0.045). Similarly, higher levels of Syncytin-2 expression were linked to an enhanced prognosis for OS (HR = 0.58, 95% CI = 0.38-0.90, P = 0.014) (**Figure 1E**). However, ERVW-1 and ERV3-1 present no significant correlation in terms of patient survival, as depicted in the corresponding Kaplan-Meier curves.

According to these results, the expression levels of Suppressyn and Syncytin-2 could function as essential indicators for AML diagnosis and prognosis.

### 3.2 The link between Suppressyn and Syncytin-2 expression with clinical characteristics

**Figure 2:**
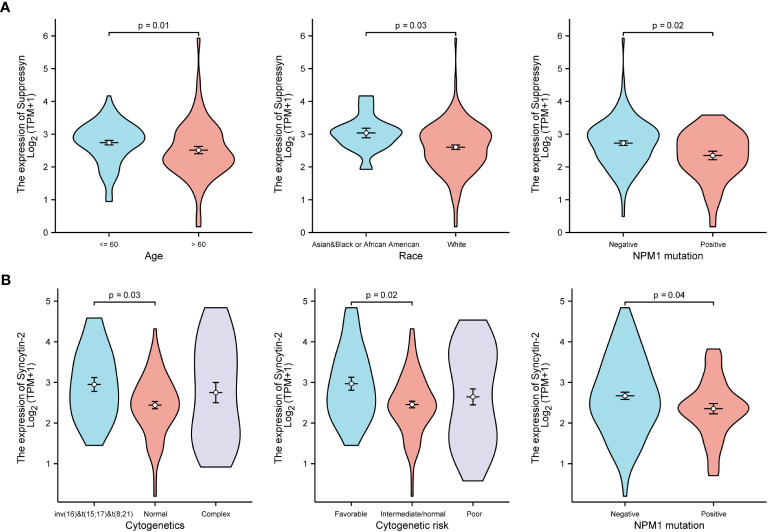
Associations between Suppressyn, Syncytin-2 expressions and clinicopathological characteristics, sourced from TCGA-AML cohorts. **(A)** Suppressyn expression in relation to ages, race, and NPM1 mutation in AML. **(B)** Syncytin-2 expression in relation to cytogenetics risk stratifications and NPM1 mutation in AML.

TCGA cohors were used to investigate how different clinical features of AML samples (**Table 1**) influenced the transcription of the two DE-HERVs. For Suppressyn, we observed statistically significant differences in the following categories: age groups (<= 60 vs > 60, P = 0.01), race groups (Asian and Black or African American vs White, P = 0.03), and NPM1 mutation (negative vs positive, P = 0.02) (**Figure 2A**). When dividing Syncytin-2 between high and low expression groups, significant statistical differences were found between subgroups in terms of cytogenetics (inv(16) & t(8;21) & t(15;17) vs normal, P = 0.03), cytogenetics risk (favorable vs intermediate/normal, P = 0.02), and NPM1 mutation (negative vs positive, P = 0.04) (**Figure 2B**). These findings supported that the transcription levels of Suppressyn and Syncytin-2 are associated with specific clinical features of AML, offering valuable insights into the potential involvement of these HERVs.

**Table 1:** Clinical features of patients with AML sourced from TCGA.

Characteristics	Suppressyn, n (%)			Syncytin-2, n (%)		
	Low	High	P value	Low	High	P value
**n**	75	75		75	75	
**Gender**			0.622218264			0.25028633
Female	32 (21.3%)	35 (23.3%)		37 (24.7%)	30 (20%)	
Male	43 (28.7%)	40 (26.7%)		38 (25.3%)	45 (30%)	
**Race**			0.087065416			0.97895236
Asian & Black or African American	4 (2.7%)	10 (6.7%)		7 (4.7%)	7 (4.7%)	
White	71 (47.7%)	64 (43%)		68 (45.6%)	67 (45%)	
**Age**			**0.001671227**			**0.031508**
<= 60	34 (22.7%)	53 (35.3%)		37 (24.7%)	50 (33.3%)	
> 60	41 (27.3%)	22 (14.7%)		38 (25.3%)	25 (16.7%)	
**WBC count (×10^9/L)**			0.08499266			0.4598313
<= 20	33 (22.1%)	43 (28.9%)		36 (24.2%)	40 (26.8%)	
> 20	42 (28.2%)	31 (20.8%)		39 (26.2%)	34 (22.8%)	
**BM blasts (%)**			0.241988929			0.61606051
<= 20	33 (22%)	26 (17.3%)		28 (18.7%)	31 (20.7%)	
> 20	42 (28%)	49 (32.7%)		47 (31.3%)	44 (29.3%)	
**PB blasts (%)**			0.25232333			0.25232333
<= 70	32 (21.3%)	39 (26%)		32 (21.3%)	39 (26%)	
> 70	43 (28.7%)	36 (24%)		43 (28.7%)	36 (24%)	
**Cytogenetic risk**			0.601456489			0.07425345
Favorable	14 (9.5%)	16 (10.8%)		10 (6.8%)	20 (13.5%)	
Intermediate/normal	44 (29.7%)	38 (25.7%)		47 (31.8%)	35 (23.6%)	
Poor	16 (10.8%)	20 (13.5%)		17 (11.5%)	19 (12.8%)	
**FAB classifications**			**0.013352135**			**0.00385272**
M0 & M1 & M2	36 (24.3%)	52 (35.1%)		43 (29.1%)	45 (30.4%)	
M3	6 (4.1%)	8 (5.4%)		2 (1.4%)	12 (8.1%)	
M4	20 (13.5%)	9 (6.1%)		17 (11.5%)	12 (8.1%)	
M5	11 (7.4%)	4 (2.7%)		12 (8.1%)	3 (2%)	
M6	2 (1.4%)	0 (0%)		0 (0%)	2 (1.4%)	
**Cytogenetics**			0.438203237			0.14077621
Normal	38 (28.4%)	31 (23.1%)		38 (28.4%)	31 (23.1%)	
inv(16) & t(15;17) & t(8;21)	12 (9%)	13 (9.7%)		8 (6%)	17 (12.7%)	
+8 & del(5) & del(7) & t(9;11) & Complex	17 (12.7%)	23 (17.2%)		20 (14.9%)	20 (14.9%)	
**FLT3 mutation**			0.772014679			0.5168341
Negative	52 (35.6%)	49 (33.6%)		53 (36.3%)	48 (32.9%)	
Positive	22 (15.1%)	23 (15.8%)		21 (14.4%)	24 (16.4%)	
**IDH1 R132 mutation**			0.771512451			0.77151245
Negative	67 (45.3%)	68 (45.9%)		67 (45.3%)	68 (45.9%)	
Positive	7 (4.7%)	6 (4.1%)		7 (4.7%)	6 (4.1%)	
**IDH1 R140 mutation**			0.078717486			0.07871749
Negative	66 (44.6%)	70 (47.3%)		66 (44.6%)	70 (47.3%)	
Positive	9 (6.1%)	3 (2%)		9 (6.1%)	3 (2%)	
**IDH1 R172 mutation**			0.46463152			0.48846312
Negative	75 (50.7%)	71 (48%)		73 (49.3%)	73 (49.3%)	
Positive	0 (0%)	2 (1.4%)		2 (1.4%)	0 (0%)	
**RAS mutation**			1			0.73089229
Negative	71 (47.7%)	70 (47%)		70 (47%)	71 (47.7%)	
Positive	4 (2.7%)	4 (2.7%)		5 (3.4%)	3 (2%)	
**NPM1 mutation**			0.18110141			**0.03345724**
Negative	55 (36.9%)	61 (40.9%)		53 (35.6%)	63 (42.3%)	
Positive	20 (13.4%)	13 (8.7%)		22 (14.8%)	11 (7.4%)	
**OS event**			0.124214914			**0.02637985**
Alive	22 (14.7%)	31 (20.7%)		20 (13.3%)	33 (22%)	
Dead	53 (35.3%)	44 (29.3%)		55 (36.7%)	42 (28%)	

n, number of patients; WBC, white blood cell; BM, bone marrow; PB, peripheral blood; FAB, French-American-British. Bold values are indicated typical factors in each AML samples.

### 3.3 Prognostic value of Suppressyn and Syncytin-2 in AML

**Figure 3:**
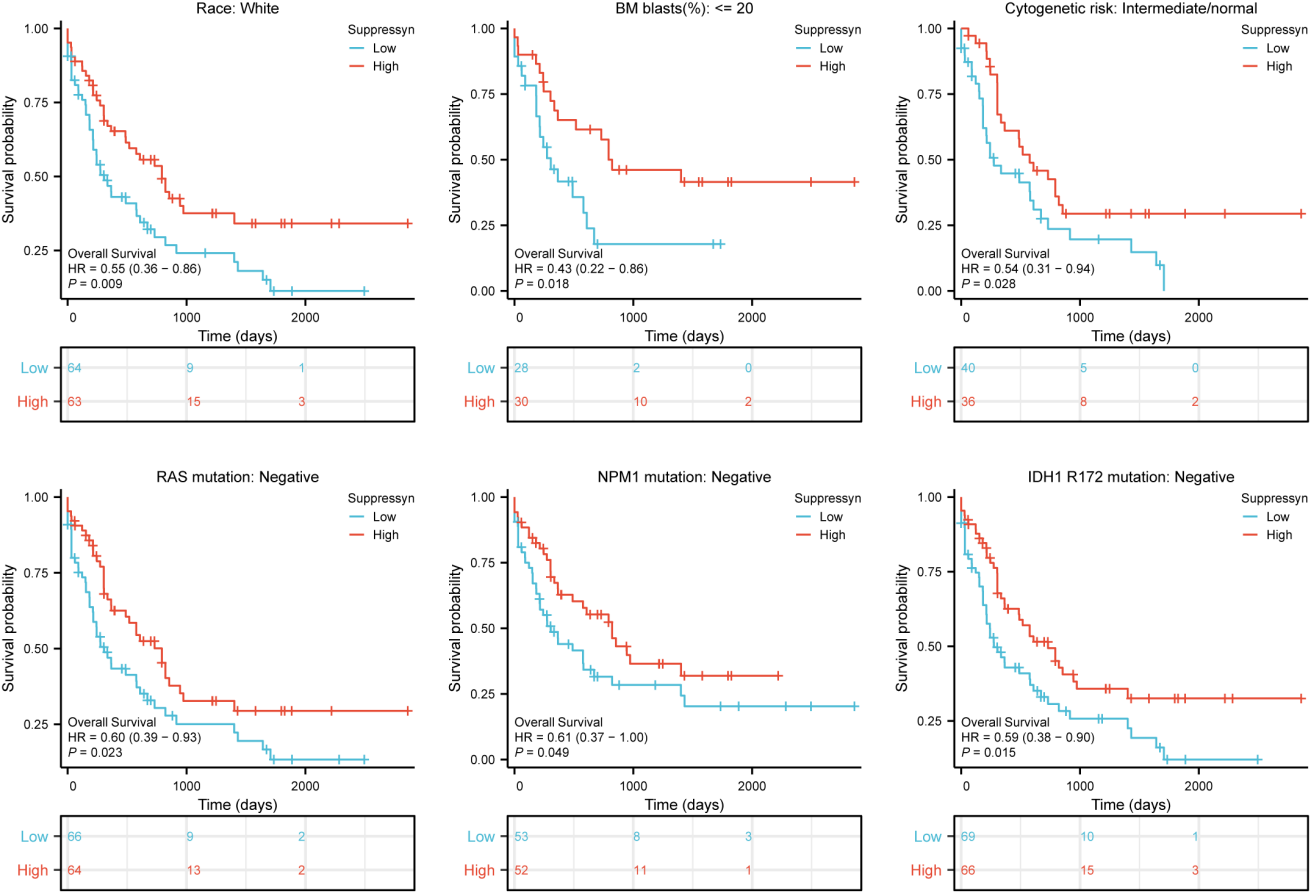
Based on Kaplan-Meier analysis, the prognostic values (OS survival curves) of Suppressyn in AML were evaluated in various clinical subgroups of races (white), BM blasts (<= 20%), cytogenetic risks (intermediate/normal), RAS mutation (negative), NPM1 mutation (negative) and IDH1 mutation (R172 negative).

Furthermore, we evaluated their prognostic value in different subgroups of AML based on OS. Our findings showed that high Suppressyn expression consistently linked to favorable outcomes in subgroups, including race (white, HR = 0.55, 95% CI = 0.36-0.86, P = 0.009), BM blasts (<= 20%, HR = 0.43, 95% CI = 0.22-0.86, P = 0.018), cytogenetic risks (intermediate/normal, HR = 0.54, 95% CI = 0.31-0.94, P = 0.028), RAS mutation (negative, HR = 0.60, 95% CI = 0.39-0.93, P = 0.023), NPM1 mutation (negative, HR = 0.61, 95% CI = 0.37-1.00, P = 0.049) and IDH1 mutation (R172 negative, HR = 0.59, 95% CI = 0.38-0.90, P = 0.015) (**Figure 3**).

**Figure 4:**
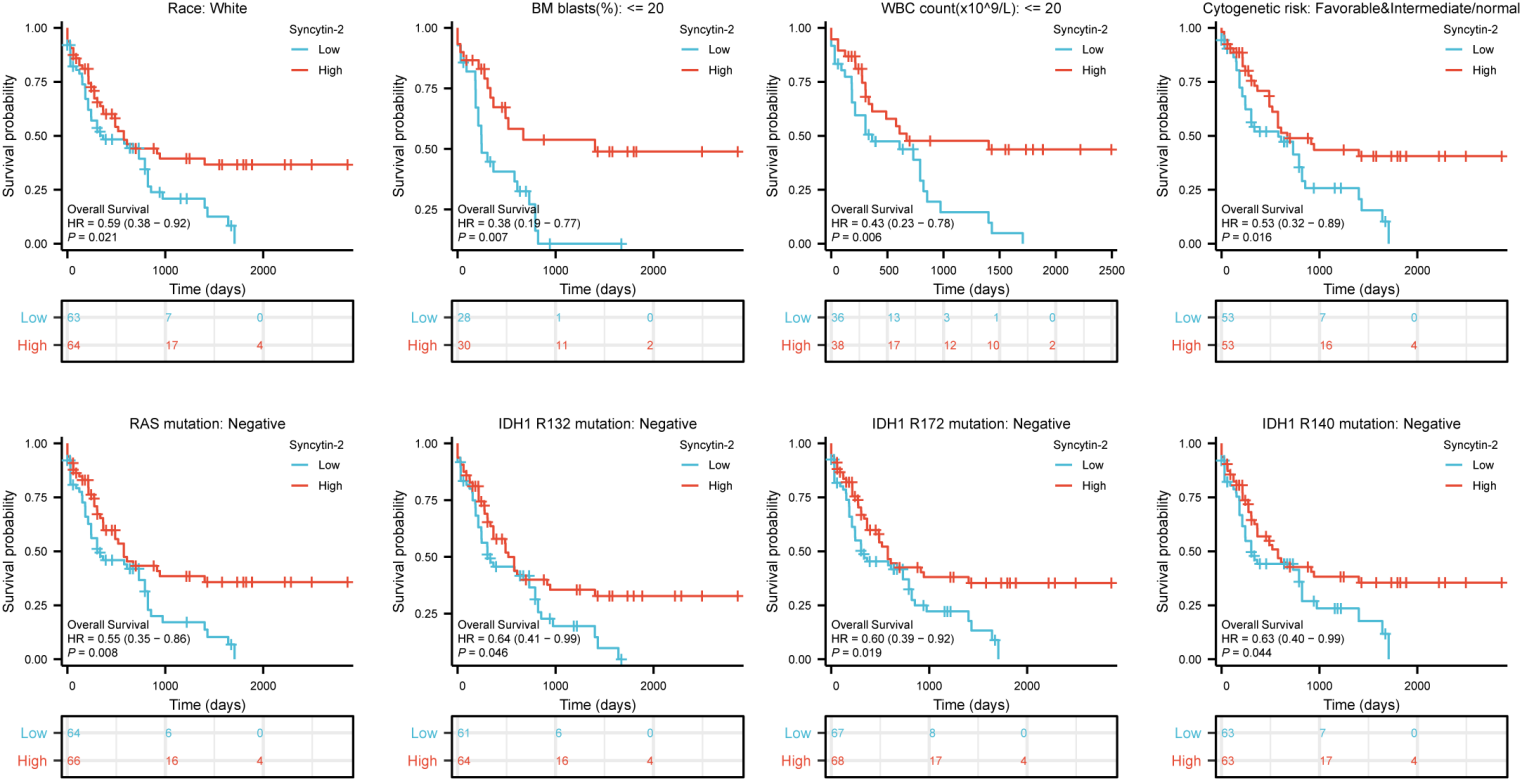
Based on Kaplan-Meier analysis, the prognostic values (OS survival curves) of Syncytin-2 in AML were evaluated in various clinical subgroups of races (white), BM blasts (<= 20%), WBC count(<= 20%), cytogenetic risks (Favorable & intermediate/normal), RAS mutation (negative), IDH1 mutation (R132 negative), IDH1 mutation (R172 negative) and IDH1 mutation (R140 negative).

Likewise, high Syncytin-2 expression linked to better survival outcomes in AML subgroups, including race (white, HR = 0.59, 95% CI = 0.38-0.92, P = 0.021), BM blasts (<= 20%, HR = 0.38, 95% CI = 0.19-0.77, P = 0.007), WBC counts (<= 20, HR = 0.43, 95% CI = 0.23-0.78, P = 0.006), cytogenetic risks (favorable & intermediate/normal, HR = 0.53, 95% CI = 0.32-0.89, P = 0.016), RAS mutation (negative, HR = 0.55, 95% CI = 0.35-0.86, P = 0.008), IDH1 mutation (R132 negative, HR = 0.64, 95% CI = 0.41-0.99, P = 0.046), IDH1 mutation (R172 negative, HR = 0.60, 95% CI = 0.39-0.92, P = 0.019), and IDH1 mutation (R140 negative, HR = 0.63, 95% CI = 0.40-0.99, P = 0.044) (**Figure 4**).

### 3.4 Analysis of DEGs and PPI network in AML based on Suppressyn and Syncytin-2

**Figure 5:**
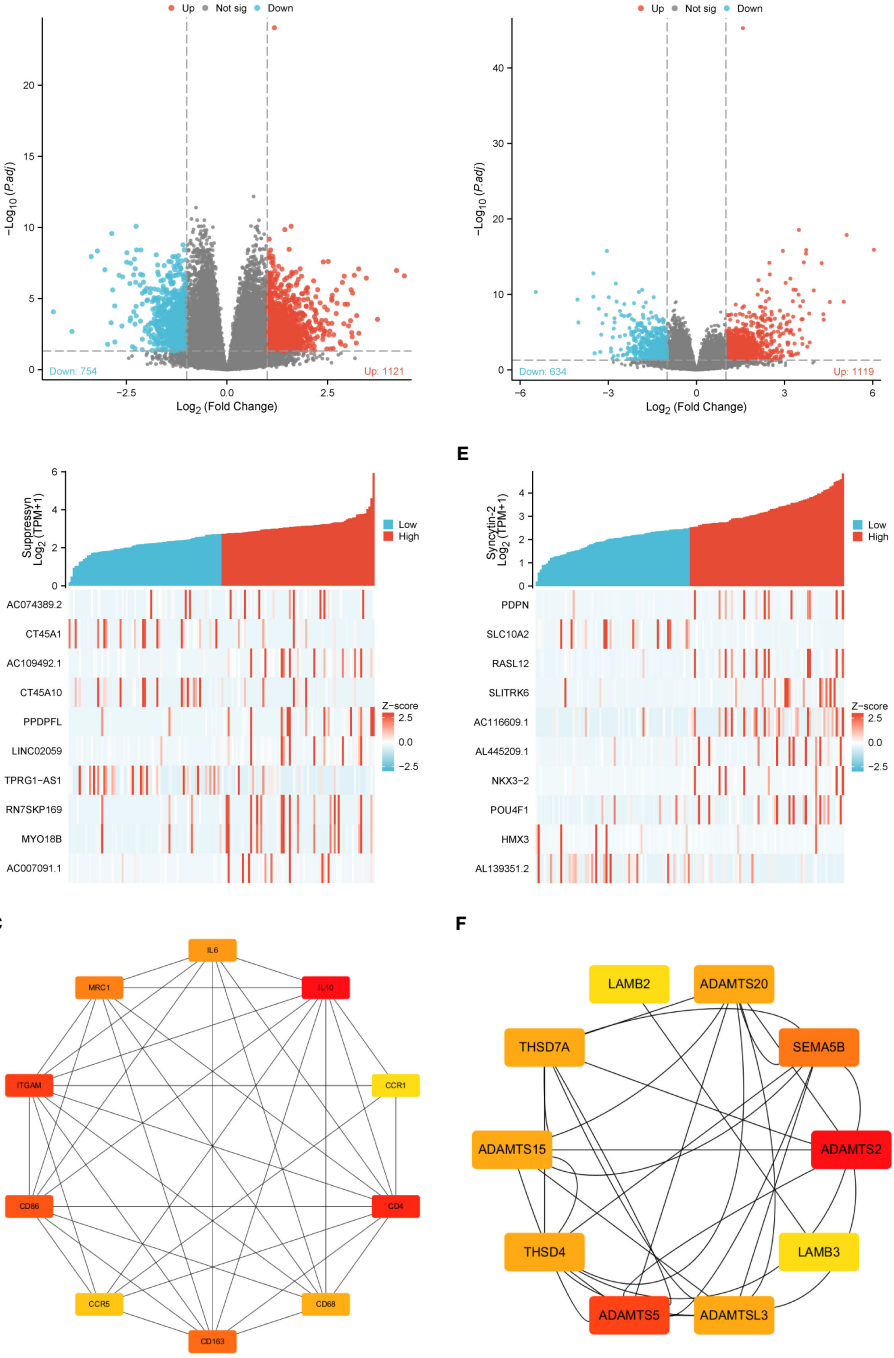
DEGs associated with Suppressyn and Syncytin-2 expression. Volcano plots of DEGs based on **(A)** Suppressyn and **(D)** Syncytin-2. The blue dots represent downregulated DEGs, while the red dots represent upregulated DEGs. Heatmap of top ten DEGs associated with **(B)** Suppressyn and **(E)** Syncytin-2. Top ten hub genes in **(C)** Suppressyn-related and **(F)** Syncytin-2-related DEGs. Higher and lower sequences are represented accordingly by red and yellow colors.

Gene expression profiles of AML cohorts exhibiting diverse expressions of two DE-HERVs enabled us to pinpoint a distinct set of DEGs. The criteria were set at |Log2-FC| > 1 and p-value< 0.05. Volcano plots displayed DEGs between high and low Suppressyn groups (**Figure 5A**). The ten most significant DEGs (AC074389.2, CT45A1, AC109492.1, CT45A10, PPDPFL, LINC02059, TPRG1-AS1, RN7SKP169, MY018B and AC007091.1) are presented in **Figure 5B** and **Supplementary Table 1**. Using the online STRING, we generated a network of protein crosstalk to explore predicted correlations between DEGs (**Supplementary Figure 1A**). Utilizing the same tool, our analysis also revealed from the PPI network an intricate group of hub genes, with the ten most significant identified as follows: IL10, CD4, ITGAM, CD86, CD163, MRC1, IL6, CD68, CCR5 and CCR1 (**Figure 5C**).

In a similar manner, we utilized volcano plots to compare DEGs in individuals with varying expression of Syncytin-2 (**Figure 5D**). We also delved deeper into the relationship between Syncytin-2 and the ten most significant DEGs, which consisted of PDPN, SLC10A2, RASL12, SLITRK6, AC116609.1, AL445209.1, NKX3-2, POU4F1, HMX3, and AL139351.2 (**Figure 5E** and **Supplementary Table 2**). Moreover, we utilized STRING to develope a PPI network, with the objective of exploring possible interactions among DEGs (**Supplementary Figure 1B**). The ten most significant hub genes were identified as ADAMTS2, ADAMTS5, SEMA5B, ADAMTS15, ADAMTSL3, THSD7A, THSD4, ADAMTS20, LAMB3, and LAMB2 (**Figure 5F**).

### 3.5 Functional enrichment analysis

**Figure 6:**
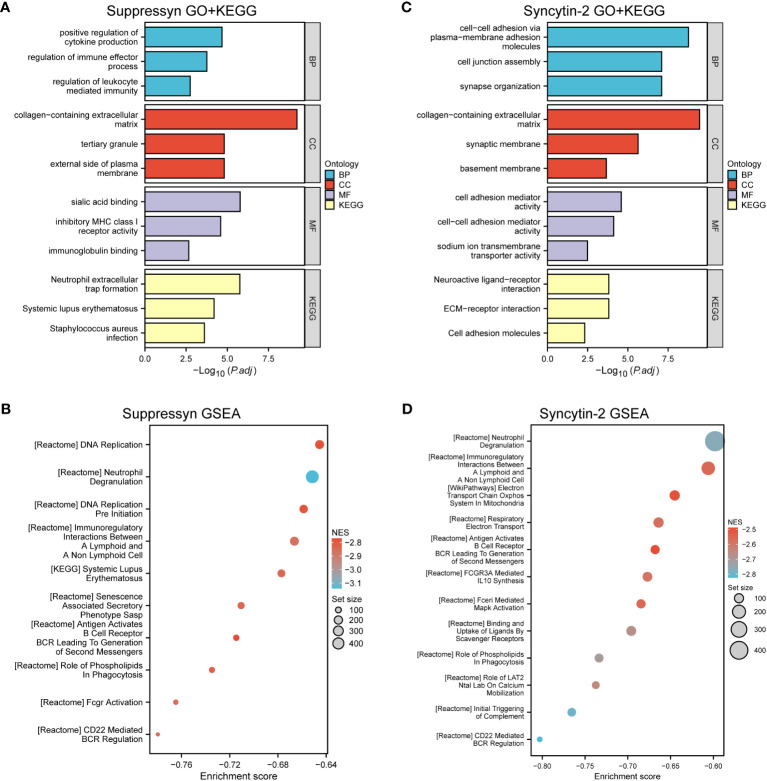
Functional Analysis of DEGs based on Suppressyn and Syncytin-2. **(A)** GO and KEGG analysis of Suppressyn-related DEGs. **(B)** Gene sets relating to Suppressyn-related DEGs were analyzed by GSEA using all canonical pathways. **(C)** GO and KEGG analysis of Syncytin-2-related DEGs. **(D)** Gene sets relating to Syncytin-2-related DEGs were analyzed by GSEA using all canonical pathways. BP, biological process; CC, cellular component; MF, molecular function; NES, normalized enrichment score.

Our analysis revealed that DEGs associated with Suppressyn showed significant enrichment in GO terms. These included the regulation of immune effector processes, collagen-containing extracellular matrix, and immunoglobulin binding. Moreover, the KEGG analysis highlighted significantly DEGs-enriched pathways, such as neutrophil extracellular trap formation (**Figure 6A** and **Supplementary Table 3**). Furthermore, we conducted Gene Set Enrichment Analysis (GSEA) using GSEA/MSigDB, which demonstrated enriched immune-associated biological processes associated with Suppressyn. These processes included immunoregulatory interactions between a lymphoid and non-lymphoid cell, antigen activation of B cell receptors, and CD22 mediated BCR regulation (**Figure 6B** and **Supplementary Table 4**).

When target switches to Syncytin-2 related DEGs, GO terms displayed a significant enrichment in response to cellular adhesion, such as molecules of plasma to membrane adhesion, collagen-containing extracellular matrix, and cell adhesion mediator activity (**Figure 6C** and **Supplementary Table 5**). Similar aspects could be found in the KEGG pathway analysis. Interestingly, GSEA analysis also discovered the potential roles of Syncytin-2 in patterns of immunoregulation (**Figure 6D** and **Supplementary Table 6**). Taken together, this functional exploration revealed both involvement of Suppressyn and Syncytin-2 in enhancing the immune phenotype in AML.

### 3.6 Correlation between Suppressyn and Syncytin-2 expression and immune infiltration

**Figure 7:**
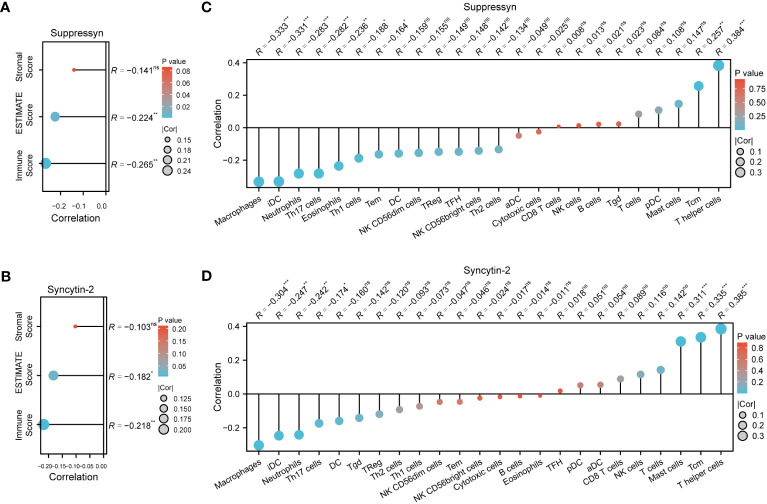
AML immune infiltration levels are correlated with expression of Suppressyn and Syncytin-2. **(A, B)** Illustration for stromal, immune, and ESTIMATE score. **(C, D)** Forrest plots showing the correlation between Suppressyn/Syncytin-2 and the immune cell levels. The dot size corresponds to the absolute values of Spearman’s correlation coefficients.

The ESTIMATE algorithm was applied to compute stromal, immune, ESTIMATE scores to determine the link between Suppressyn and Syncytin-2 and immune infiltrates in AML. Both Suppressyn and Syncytin-2 displayed a notable inverse correlation with immune scores and ESTIMATE scores, indicating a substantial influence on immune cell infiltration (**Figures 7A, B**).

In addition, we employed single-sample GSEA to demonstrate a positive link between Suppressyn with T-helper cells (r = 0.384) and Tcm (r = 0.257), while a negative link was oberved with macrophages (r = -0.333) and iDC (r = -0.331) (**Figure 7C**). With respect to Syncytin-2, we found it positively linked with multiple immune infiltrates, including T-helper cells (r = 0.385), Tcm (r = 0.335), and mast cells (r = 0.311), but a negative linke with macrophages (r = -0.304) (**Figure 7D**). All these findings were within strong significance, with P< 0.001.

**Figure 8:**
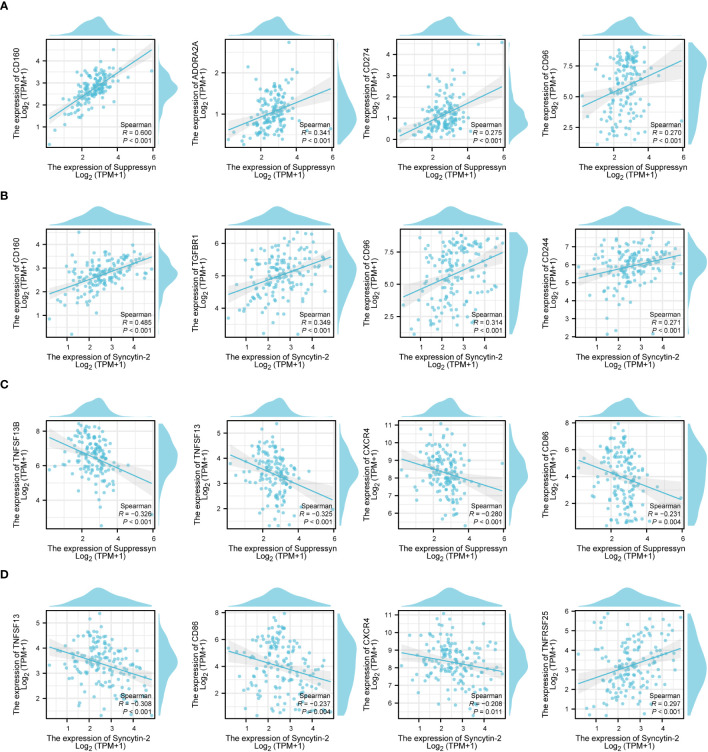
The correlation of Suppressyn and Syncytin-2 with immunomodulators in AML. **(A)** Suppressyn and immunoinhititors; **(B)** Syncytin-2 and immunoinhititors; **(C)** Suppressyn and immunostimulators; **(D)** Syncytin-2 and immunostimulators.

Following this, we explored the relationship between Suppressyn and Syncytin-2 expression and immune checkpoints, grouped by immunoinhibitors and immunostimulators. Notably, both Suppressyn and Syncytin-2 showed similar expression patterns. They were positively correlated with immunoinhibitors, such as CD160, ADORA2A, CD274, CD96, TGFBR1 and CD244 (**Figures 8A, B**), while displaying a negative correlation with immunostimulators, including TNFSF13B, TNFSF13, CXCR4 and CD86 (**Figures 8C, D**), with more details showed in **Supplementary Table 7**.

### 3.7 Construction of a prognostic (risk-score) model based on Suppressyn and Syncytin-2

**Figure 9:**
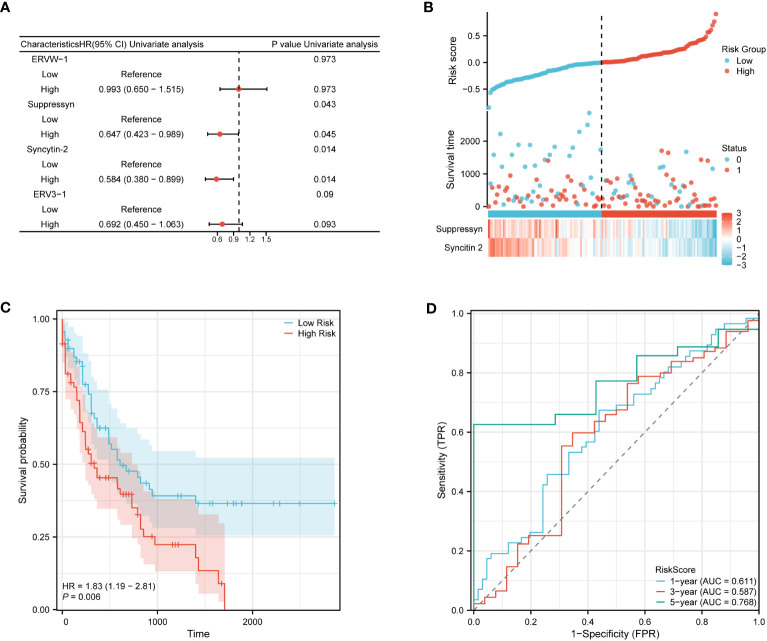
Prognostic (Risk-score) model based on Suppressyn and Syncytin-2 in TCGA cohort. **(A)** The forest map using univariate Cox regression of four DE-HERVs for OS. **(B)** Up: Patients were evenly categorized based on the median risk score threshold. The low-risk group is colored blue, while the high-risk group is colored red. Middle: Survival conditions between high and low-risk groups are depicted. Blue signifies survival, while red indicates death. Down: Expression of Suppressyn and Syncytin-2 on a heatmap. Lower risks are colored blue, while higher risks are colored red. **(C)** The Kaplan-Meier curves of OS in high- and low-risk groups. **(D)** The ROC curve analyses (at 1-, 3-, and 5-years) to verify the predictive efficiency of the risk score.

The ability to accurately classify patients as either high- or low-risk for AML progression is of utmost importance for patient management. After conducting an initial preselection step using univariate Cox regression analysis, Suppressyn and Syncytin-2 are significantly linked to patient survival (P< 0.05) (**Figure 9A**). To take into account the expression patterns and regression coefficients, we computed a risk score using the formula: Risk score = Suppressyn expression × (-0.19727) + Syncytin-2 expression × (-0.20209). According to their risk scores, AML patients sourced from the TCGA cohort were stratified as low- and high-risk groups (**Figure 9B**). Individuals in the low-risk category experienced lower mortality rates and longer survival periods compared to those in the high-risk category. According to the Kaplan-Meier curves, the high-risk category demonstrated a lower probability of survival in comparison to the low-risk category (HR = 1.83, 95% CI = 1.19 – 2.81, P = 0.006) (**Figure 9C**). The prognostic accuracy for OS stood at 0.611 after 1 year, 0.587 after 3 years, and 0.768 after 5 years based on time-dependent ROC analysis (**Figure 9D**). These results highlight the potential of the Suppressyn and Syncytin-2 -based risk-scoring model as a valuable tool for predicting AML patient outcomes.

## 4 Discussion

Previous studies have indicated that HERV is detectable in both normal and pathological conditions, with varying degrees of expression thresholds(Matteucci et al., 2018; Larouche et al., 2020; Kassiotis, 2023; Stricker et al., 2023b). High-throughput RNA sequencing of datasets including AML has revealed a comprehensive screening platform to identify potential HERV families and elements(Tokuyama et al., 2018; Deniz et al., 2020; Engel et al., 2021; Ng et al., 2023; Stricker et al., 2023a). However, few studies have provided direct evidence of specific HERVs that serve as clearer biomarkers or therapeutic targets, and this issue requires further investigation and resolution.

In our study, we made a novel observation regarding the high expression of Suppressyn (ERVH48-1, HGCN: 17216) and Syncytin-2 (ERVFRD-1, HGCN: 33823) in AML, which showed significant survival differences. This finding is particularly exciting because Suppressyn and Syncytin-2, previously known for their essential role in the merging of villous trophoblast with the syncytiotrophoblast (also proved by **Figure 6**), now appear to play a crucial role in AML as well (Denner, 2016). While cell-cell fusion processes similar to viral infection and cancer metastasis are relatively rare in hematological malignancies, this study highlights their potential importance in AML. It is worth noting that we excluded syncytin-1 (ERVW-1, HGCN: 13525) from our subsequent analysis due to its lack of clinical significance. An additional reason for excluding syncytin-1 from our analysis is that epidemiological associations should go beyond simply supporting biological hypotheses. Instead, they should align with the existing knowledge regarding practical outcomes and pathogenesis of AML.

Next, we discovered that Suppressyn and Syncytin-2 exhibited significant diagnostic value in AML patients, as indicated by high ROC levels (**Figure 1**). These two HERVs also served as promising prognostic biomarkers in AML, supported by strong correlation with favorable outcomes such as lower cytogenetic risks and absence of mutations in NPM1. Mutation of NPM1 generally coincides with FLT3-ITD, DNMT3A or IDH1/2, leading to inferior recovery and OS in AML(Papaemmanuil et al., 2016; Dunlap et al., 2019). Real-world observation studies have suggested that AML patients with lower copies of NPM1/mut tend to have a favorable prognosis(Döhner et al., 2017), which aligns with the favorable clinical outcomes based on our findings in higher Suppressyn groups (**Figure 3**). Additionally, the prognostic influence of IDH mutations can differ depending on the specific location of the mutation at diagnosis, such as IDH1 R132, IDH2 R140/R172(Pollyea et al., 2023). Thus, presence of non-mutation IDH alongside Suppressyn could be a potentially underlying biological factor that affects patients, particularly individuals with IDH-mutated AML after hematopoietic stem cell transplantation (HSCT)(Bill et al., 2023). Further investigation into how candidate HERVs correlate with cytogenetic analysis and molecular markers, including NPM1 and IDH, can help refine current prognostics groups, particularly in AML patients with a normal karyotype.

We also investigated the potential influence of Suppressyn and Syncytin-2 on tumor microenvironment. The immune aspects of the bone marrow microenvironment play a crucial role in the biology of AML, influencing therapy response and patient survival. In this context, we made an exciting discovery that higher expression of Suppressyn and Syncytin-2 may function as, or at least closely related to, favorable immunoregulators.

First, ESTIMATE algorithm revealed a significantly negative correlation between Suppressyn/Syncytin-2 and immune scores (**Figure 7**). Higher levels of either Suppressyn or Syncytin-2 were associated with low immune risk scores, which are known to be linked to prolonged EFS (events-free survival) and OS (Wang et al., 2021). In addition, we analyzed immune infiltrating cells, another key factor contributing to the heterogeneous outcomes of AML patients(Johnson et al., 2022). The results indicated that these two HERVs might exhibit a distinctive immune signature. For instance, activation of T cell subsets, especially T helper cells, was commonly observed in both Suppressyn and Syncytin-2, suggesting that although they no longer possess infectious activities, the immune system still recognizes the HERV-env proteins as virus components. Moreover, we observed a higher abundance of mast cell infiltration in cases with elevated levels of Syncytin-2 (**Figure 7D**). Similar evidence supporting better clinical outcomes associated with these HERVs has been found not only in AML by computational analysis(Zeng et al., 2021), but also in research-based study in lung adenocarcinoma(Fan et al., 2023). It is worth noting that our findings also confirmed the poor prognostic impact of macrophage infiltration(Xu et al., 2020), which negatively correlated with Suppressyn and/or Syncytin-2 in AML patients. In summary, these findings are largely in line with published data, indicating a protective potential of Suppressyn and Syncytin-2 in AML.

Therefore, our study identified the active potential of both Suppressyn and Syncytin-2 in pathways and targets associated with immunotherapy. This highlights at least two strategies for leveraging their anti-leukemic effects. As an initial approach, HERVs are exploited for their inherent properties as targets in myeloid malignancies with low mutational burdens(Saini et al., 2020). Mutations occurring in HERV-derived sequences may generate neoantigens perceived as foreign epitopes, thereby amplifying immune responses against AML cells. The strong link between tumor mutation burden and treatment responses has spurred investigations into the hot topic of immune checkpoint blockade(Yarchoan et al., 2017; Legrand et al., 2018). Targeting specific HERV-derived antigens through passive and active immune stimulation could be another viable approach to elicit an adaptive immune response against cells expressing HERVs(Kraus et al., 2014; Russ and Iordanskiy, 2023). In this regard, identifying HERV tumor-specific antigens for the development of broad-spectrum anticancer strategies holds significant therapeutic potential. Expanding upon this research and executing it in clinical studies on immunotherapy will significantly enhance our understanding regarding the importance of HERVs as targets for adaptive immune therapy.

According to the findings of this study, both Suppressyn and Syncytin-2 hold significant importance in AML patients. The exact mechanism by which these two factors operate, whether through divergent pathways or in a synergistic manner, and whether their function is influenced by multiple factors associated with evolutionary conservation or oncogenic mutations, remains uncertain. We believe the potential for targeted therapy discussed earlier in this article makes future research challenging yet thrilling.

## 5 Conclusions

In conclusion, our study revealed crucial roles of both Suppressyn and Syncytin-2 in AML. Our research revealed diverse expression in these HERVs between AML samples and controls, and their expression was correlated with specific clinical characteristics of the disease. Additionally, varied expressions of Suppressyn and Syncytin-2 could be linked to multiple immune infiltrates and immune checkpoints, highlighting their potential involvement in immune regulation and immune response in AML. These discoveries enhance our comprehension of the molecular landscape and immunological implications of HERVs in AML, suggesting their possibility of serving as prognostic and diagnostic markers, as well as potentially becoming future therapeutic targets.

## Data availability statement

The original contributions presented in the study are included in the article/**Supplementary Material**. Further inquiries can be directed to the corresponding author.

## Author contributions

JS: Formal analysis, Investigation, Methodology, Writing – original draft. XW: Data curation, Investigation, Methodology, Software, Writing – original draft. XX: Investigation, Formal analysis, Writing – original draft. CF: Data curation, Investigation, Writing – review & editing. LS: Conceptualization, Funding acquisition, Project administration, Resources, Supervision, Visualization, Writing – review & editing.
