# Supplementary material for: Endogenous retroviruses Suppressyn and Syncytin-2 as innovative prognostic biomarkers in Acute Myeloid Leukemia
Source: Front Cell Infect Microbiol. 2024 Jan 11;13:1339673. doi: 10.3389/fcimb.2023.1339673 (PMC10808309; doi:10.3389/fcimb.2023.1339673)
Supplement: Supplementary file 1 [file DataSheet_1.pdf]

Supplementary Information

Article Title: Endogenous Retroviruses Suppressyn and Syncytin-2 as Innovative Prognostic Biomarkers in Acute Myeloid Leukemia

Author names: Jiaxin Shen, Xiaofen Wen, Xueyang Xing, Claudio Fozza, and Leonardo Antonio Sechi*

Corresponding author: Leonardo Antonio Sechi, sechila@uniss.it

Department of Biomedical Sciences; Unit of Microbiology and Virology, Agency of University Hospital (AOU), 07100 Sassari, Italy.

Supplemental Fig S1. (A) Protein–protein interaction network of Suppressyn-related DEGs. (B) Protein–protein interaction network of Syncytin-2-related DEGs.

A

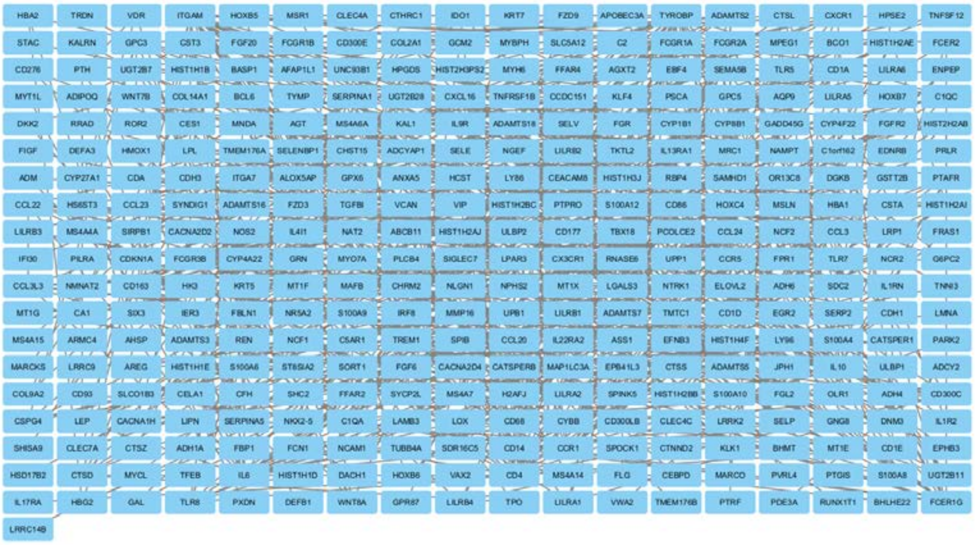

B

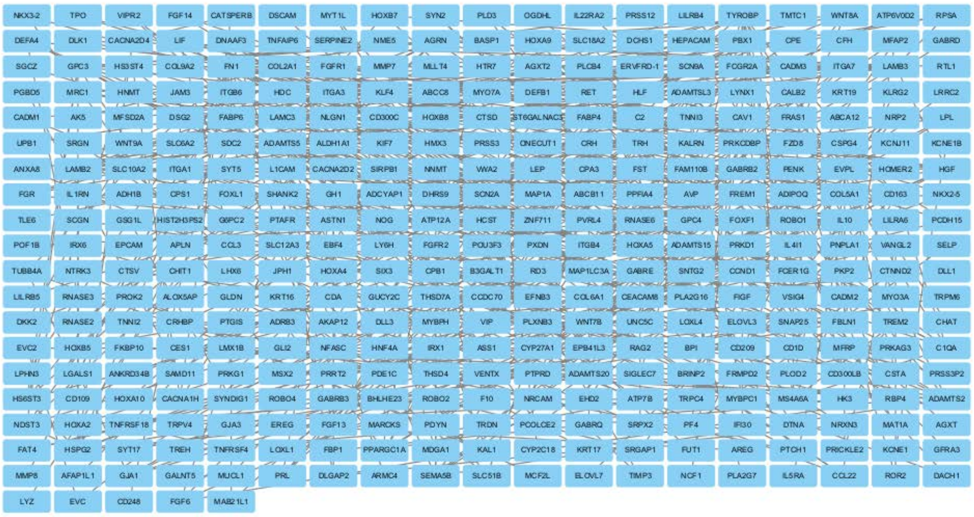

Supplementary table S2. Top 10 Suppressyn related DEGs.

gene_name	id	baseMean	log2FoldChange	lfcSE	stat	pvalue	padj	gene_type
AC074389.2	ENSG00000231476.1	4.343586	4.407455	0.738116	5.971222	2.35E-09	2.56021E-07	lncRNA
CT45A10	ENSG00000269586.7	17.0008	-3.85054	1.047734	-3.67511	0.000238	0.002098035	protein_coding
AC109492.1	ENSG00000249061.1	2.812098	4.211843	0.682334	6.172698	6.71E-10	1.07369E-07	lncRNA
CT45A10	ENSG00000269586.7	17.0008	-3.85054	1.047734	-3.67511	0.000238	0.002098035	protein_coding
PPDPFL	ENSG00000168333.14	8.928729	3.738872	0.876767	4.264386	2E-05	0.000293862	protein_coding
LINC02059	ENSG00000250544.2	4.190359	3.462307	0.5874	5.894288	3.76E-09	3.67485E-07	lncRNA
TPRG1-AS1	ENSG00000234076.1	9.607159	-3.37123	0.508402	-6.63102	3.33E-11	1.1233E-08	lncRNA
RN7SKP169	ENSG00000223056.1	2.38675	3.278761	0.915041	3.583185	0.000339	0.002780255	misc_RNA
MYO18B	ENSG00000133454.16	344.2343	3.272097	0.525778	6.223348	4.87E-10	8.2621E-08	protein_coding
AC007091.1	ENSG00000223838.2	1.9177	3.223579	0.727801	4.429202	9.46E-06	0.000160206	lncRNA

Abbreviations: DEGs, differentially expressed genes; p.adj, adjusted p value.

Supplementary table S3. Top 10 Syncytin-2 related DEGs.

gene_name	id	baseMean	log2FoldChange	lfcSE	stat	pvalue	padj	gene_type
PDPN	ENSG00000162493.16	29.58699	6.038652	0.647908	9.320234	1.16E-20	1.22E-16	protein_coding
SLC10A2	ENSG00000125255.7	29.64237	-5.46682	0.718154	-7.61233	2.69E-14	4.7E-11	protein_coding
RASL12	ENSG00000103710.11	34.30219	5.112194	0.520673	9.818434	9.38E-23	1.31E-18	protein_coding
SLITRK6	ENSG00000184564.11	14.95891	5.011537	0.69974	7.161999	7.95E-13	9.01E-10	protein_coding
AC116609.1	ENSG00000223751.1	7.238773	4.540625	0.63589	7.140588	9.29E-13	9.99E-10	lncRNA
AL445209.1	ENSG00000271776.1	12.13213	4.331444	0.699702	6.190409	6E-10	2.19E-07	lncRNA
NKX3-2	ENSG00000109705.8	5.744143	4.318163	0.660614	6.536595	6.29E-11	4.12E-08	protein_coding
POU4F1	ENSG00000152192.8	203.7236	4.26086	0.486053	8.76625	1.85E-18	7.04E-15	protein_coding
HMX3	ENSG00000188620.11	9.620258	-4.04907	0.557017	-7.2692	3.62E-13	4.74E-10	protein_coding
AL139351.2	ENSG00000286179.1	20.52487	-4.01711	0.66647	-6.02745	1.67E-09	5.13E-07	lncRNA

Abbreviations: DEGs, differentially expressed genes; p.adj, adjusted p value.

Supplementary table S4. Gene Ontology and KEGG pathway functional enrichment for Suppressyn related DEGs.

ONTOLOGY	ID	Description	GeneRatio	BgRatio	pvalue	p.adjust	qvalue
BP	GO:0001819	positive regulation of cytokine production	47/731	475/18800	4.47E-09	2.08E-05	1.83E-05
BP	GO:0002697	regulation of immune effector process	35/731	353/18800	4.1E-07	0.000174	0.000152
BP	GO:0002703	regulation of leukocyte mediated immunity	24/731	236/18800	1.76E-05	0.00177	0.001552
CC	GO:0062023	collagen-containing extracellular matrix	51/764	429/19594	1.34E-12	5.86E-10	5.31E-10
CC	GO:0070820	tertiary granule	23/764	164/19594	1.05E-07	1.53E-05	1.38E-05
CC	GO:0009897	external side of plasma membrane	43/764	455/19594	8.52E-08	1.53E-05	1.38E-05
MF	GO:0033691	sialic acid binding	10/733	22/18410	3.95E-09	1.64E-06	1.44E-06
MF	GO:0032396	inhibitory MHC class I receptor activity	7/733	12/18410	1.03E-07	2.51E-05	2.2E-05
MF	GO:0019865	immunoglobulin binding	7/733	24/18410	2.95E-05	0.002166	0.001895
KEGG	hsa04613	Neutrophil extracellular trap formation	30/392	190/8164	6.08E-09	1.74E-06	1.61E-06
KEGG	hsa05322	Systemic lupus erythematosus	22/392	136/8164	4.43E-07	6.34E-05	5.88E-05
KEGG	hsa05150	Staphylococcus aureus infection	17/392	96/8164	2.57E-06	0.000245	0.000227

Abbreviations: BP, biological processes; CC, subcellular localizations; MF, molecular functions; KEGG, Kyoto Encyclopedia of Genes and Genomes; DEGs, differentially expressed genes.

Supplementary table S5. Gene set enrichment analysis for Suppressyn related DEGs.

ID	setSize	Enrichment Score	NES	pvalue	p.adjust	qvalue	rank
REACTOME_DNA_REPLICATION	187	-0.64549	-2.78481	1E-10	2.05E-09	1.16E-09	9471
REACTOME_NEUTROPHIL_DEGRANULATION	478	-0.65151	-3.14006	1E-10	2.05E-09	1.16E-09	8278
REACTOME_DNA_REPLICATION_PRE_INITIATION	159	-0.6587	-2.78789	1E-10	2.05E-09	1.16E-09	9471
REACTOME_IMMUNOREGULATORY_INTERACTIONS_BETWEEN A_LYMPHOID_AND_A_NON_LYMPHOID_CELL	186	-0.66634	-2.87197	1E-10	2.05E-09	1.16E-09	5086
KEGG_SYSTEMIC_LUPUS_ERYTHEMATOSUS	136	-0.67715	-2.83928	1E-10	2.05E-09	1.16E-09	7589
REACTOME_SENESCENCE_ASSOCIATED_SECRETORY_PHENOTYPE_SASP	111	-0.71055	-2.84123	1E-10	2.05E-09	1.16E-09	8367
REACTOME_ANTIGEN_ACTIVATES_B_CELL_RECEPTOR_BCR_ LEADING_TO_GENERATION_OF_SECOND_MESSENGERS	86	-0.71457	-2.77019	1E-10	2.05E-09	1.16E-09	5718
REACTOME_ROLE_OF_PHOSPHOLIPIDS_IN_PHAGOCYTOSIS	82	-0.73474	-2.82013	1E-10	2.05E-09	1.16E-09	6034
REACTOME_FCGR_ACTIVATION	69	-0.7647	-2.85491	1E-10	2.05E-09	1.16E-09	6383
REACTOME_CD22_MEDIATED_BCR_REGULATION	61	-0.7796	-2.86144	1E-10	2.05E-09	1.16E-09	5718

Abbreviations: GSEA, gene set enrichment analysis; BP, biological processes; CC, subcellular localizations; MF, molecular functions.

Supplementary table S6. Gene Ontology and KEGG pathway functional enrichment for Syncytin-2 related DEGs.

ONTOLOGY	ID	Description	GeneRatio	BgRatio	pvalue	p.adjust	qvalue
BP	GO:0098742	cell-cell adhesion via plasma-membrane adhesion molecules	42/779	279/18800	3.77E-13	1.73E-09	1.52E-09
BP	GO:0034329	cell junction assembly	49/779	420/18800	6.2E-11	7.97E-08	7.03E-08
BP	GO:0099172	presynapse organization	10/779	52/18800	4.59E-05	0.004162	0.00367
CC	GO:0062023	collagen-containing extracellular matrix	53/805	429/19594	7.96E-13	3.5E-10	2.86E-10
CC	GO:0097060	synaptic membrane	41/805	373/19594	1.06E-08	2.33E-06	1.91E-06
CC	GO:0005604	basement membrane	16/805	95/19594	1.5E-06	0.00022	0.00018
MF	GO:0098631	cell adhesion mediator activity	15/754	64/18410	3.27E-08	2.6E-05	2.27E-05
MF	GO:0098632	cell-cell adhesion mediator activity	13/754	54/18410	1.93E-07	7.65E-05	6.7E-05
MF	GO:0015081	sodium ion transmembrane transporter activity	19/754	150/18410	1.23E-05	0.003256	0.002854
KEGG	hsa04512	ECM-receptor interaction	16/358	88/8164	1.08E-06	0.000154	0.00015
KEGG	hsa04080	Neuroactive ligand-receptor interaction	37/358	362/8164	1.11E-06	0.000154	0.00015
KEGG	hsa04514	Cell adhesion molecules	19/358	157/8164	5.29E-05	0.004885	0.004771

Abbreviations: BP, biological processes; CC, subcellular localizations; MF, molecular functions; KEGG, Kyoto Encyclopedia of Genes and Genomes; DEGs, differentially expressed genes.

Supplementary table S7. Gene set enrichment analysis for Syncytin-2 related DEGs.

ID	setSize	enrichmentScore	NES	pvalue	p.adjust	qvalue	rank
REACTOME_NEUTROPHIL_DEGRANULATION	478	-0.598	-2.76468	1E-10	6.82E-09	5.4E-09	7211
REACTOME_IMMUNOREGULATORY_INTERACTIONS_BETWEEN_A_LYMPHOID_AND_A_NON_LYMPHOID_CELL	185	-0.60598	-2.55477	1E-10	6.82E-09	5.4E-09	3629
REACTOME_RESPIRATORY_ELECTRON_TRANSPORT	103	-0.66404	-2.57347	1E-10	6.82E-09	5.4E-09	6802
REACTOME_ANTIGEN_ACTIVATES_B_CELL_RECEPTOR_BCR_LEADING_TO_GENERATION_OF_SECOND_MESSENGERS	85	-0.66804	-2.48753	1E-10	6.82E-09	5.4E-09	5087
REACTOME_FCGR3A_MEDIATED_IL10_SYNTHESIS	94	-0.67704	-2.5845	1E-10	6.82E-09	5.4E-09	4440
REACTOME_FCERI_MEDIATED_MAPK_ACTIVATION	86	-0.68458	-2.55148	1E-10	6.82E-09	5.4E-09	4858
REACTOME_BINDING_AND_UPTAKE_OF_LIGANDS_BY_SCAVENGER_RECEPTORS	97	-0.69596	-2.67046	1E-10	6.82E-09	5.4E-09	3654
REACTOME_ROLE_OF_PHOSPHOLIPIDS_IN_PHAGOCYTOSIS	81	-0.73359	-2.70748	1E-10	6.82E-09	5.4E-09	4440
REACTOME_ROLE_OF_LAT2_NTAL_LAB_ON_CALCIUM_MOBILIZATION	70	-0.73743	-2.64963	1E-10	6.82E-09	5.4E-09	3629
REACTOME_INITIAL_TRIGGERING_OF_COMPLEMENT	78	-0.76552	-2.79767	1E-10	6.82E-09	5.4E-09	3823
REACTOME_CD22_MEDIATED_BCR_REGULATION	60	-0.80293	-2.8234	1E-10	6.82E-09	5.4E-09	4440

Abbreviations: GSEA, gene set enrichment analysis; BP, biological processes; CC, subcellular localizations; MF, molecular functions.

Supplementary table S8. Correlation analysis between Suppressyn, Syncytin-2 and immune checkpoints.

Correlation analysis between Suppressyn, Syncytin-2 and immunoinhibitors

HERVs	Gene Markers	Correlation	P value
Suppressyn	CD160	0.60048	4.58E-16
	ADORA2A	0.34108	1.94E-05
	CD244	0.29558	0.0002
	CD274	0.2746	0.0007
	CD96	0.27049	0.0008
	IDO1	0.23214	0.0043
	TIGIT	0.22506	0.0056
	TGFBR1	0.17033	0.0372
	LGALS9	-0.16868	0.0391
	CD160	0.48478	4.82E-10
Syncytin-2	TGFBR1	0.34923	0.0000135
	CD96	0.31365	0.0001
	CD244	0.27066	0.0008
	ADORA2A	0.21125	0.0095
	TIGIT	0.21129	0.0096
	IL10	-0.20759	0.0108
	CD274	0.20456	0.012

Correlation analysis between Suppressyn, Syncytin-2 and immunostimulators

HERVs	Gene Markers	Correlation	P value
Suppressyn	TNFSF13	-0.30836	0.0001
	TNFRSF25	0.2972	0.0002
	TMIGD2	0.27845	0.0006
	PVR	0.25045	0.002
	ICOSLG	0.24518	0.0026
	TNFSF18	0.24033	0.0031
	CD86	-0.23736	0.0035
	IL2RA	0.22873	0.005
	CD80	0.22752	0.0051
	TNFSF4	0.22066	0.0067
	CXCR4	-0.20755	0.0109
	RAET1E	0.18619	0.0225
	TNFRSF18	0.16336	0.0458
	TNFRSF17	0.163	0.0464
	CD40LG	0.16247	0.0471
Syncytin-2	CD40LG	0.16247	0.0471
	CD80	0.22752	0.0051
	CD86	-0.23736	0.0035
	CXCR4	-0.20755	0.0109

	ICOSLG	0.24518	0.0026
	IL2RA	0.22873	0.005
	PVR	0.25045	0.002
	RAET1E	0.18619	0.0225
	TMIGD2	0.27845	0.0006
	TNFRSF17	0.163	0.0464
	TNFRSF18	0.16336	0.0458
	TNFRSF25	0.2972	0.0002
	TNFSF13	-0.30836	0.0001
	TNFSF18	0.24033	0.0031
	TNFSF4	0.22066	0.0067
